# Validation of Invasive Area for Predicting Sentinel Node Status and Survival in Primary Cutaneous Melanoma

**DOI:** 10.1245/s10434-025-17442-2

**Published:** 2025-05-23

**Authors:** Sophie E. Orme, Mark Bamford, Marie O’Riordan, Philip D. Da Forno, Andrew Snelling, Martin J. Heaton, Rachael Stanley, Roxanne Brunton-Sim, Marc D. Moncrieff, Gerald Saldanha

**Affiliations:** 1 Norfolk and Norwich University Hospitals NHS Trust, Norwich, UK; 2 Norwich Medical School, University of East Anglia, Norwich, UK; 3 University Hospitals of Leicester NHS Trust, Leicester, UK

## Abstract

**Background:**

Two-dimensional histologic features have demonstrated independent prognostic value for survival in primary cutaneous melanoma, but their predictive value for sentinel node (SN) status has yet to be validated. We aimed to demonstrate the predictive value of the previously described calculated tumor area (CTA), and the novel Simplified Breslow Area (SBA), for SN metastasis and survival.

**Materials and Methods:**

A total of 177 primary melanomas were assessed for standard histological characteristics, maximum invasive width (IW) of the primary tumor and CTA. We simplified CTA measurement by transforming IW with Breslow thickness (BT) [ln(IW) + ln (BT)], yielding SBA. Multivariate analysis was undertaken to assess the performance of CTA and SBA, respectively, as independent predictors of both SN status and survival outcomes.

**Results:**

The SN + rate was 18.1% (32/177). The median CTA for SN−patients was 3.2 mm^2^ (IQR 1.2–10.9) compared with 6.7 mm^2^ (IQR 4.2–26.9) for SN + patients (*p* < 0.01). Maximum threshold analysis identified an optimal CTA cutoff point of 6.3 mm^2^ for disease-specific (DSS) [HR 1.01 (1.00–1.02); *p* = 0.008], distant metastasis-free [HR 1.01 (1.00–1.02); *p* = 0.005], and disease-free survival [HR 1.01 (1.00–1.02); *p* = 0.005]. The 5-year DSS for low-risk CTA tumors was 91.2% versus 61.3% for high-risk tumors. Cox regression showed CTA [HR 3.5 (1.21–10.81); *p* = 0.021] and ulceration status (US) were independent predictors of DSS. Similar results were obtained for SBA, which, on multivariate analysis, was the single most important predictor of SN status outperforming lymphovascular invasion, US, and BT.

**Conclusions:**

The two-dimensional histologic features CTA and SBA are independently prognostic for survival in primary cutaneous melanoma, and SBA may be a better predictor of SN status than BT.

**Supplementary Information:**

The online version contains supplementary material available at 10.1245/s10434-025-17442-2.

The introduction of effective adjuvant therapy for cutaneous melanoma, as well as the results of the Multicenter Selective Lymphadenectomy Trial II (MSLT-2) and German Dermatologic Cooperative Oncology Group (DeCOG) trials, has led to a paradigm shift in international opinion on the primary role of sentinel node biopsy (SNB).^1–5^ Crucially, the determination of sentinel node (SN) status has become a key factor influencing access to adjuvant systemic therapies by identifying micrometastatic stage III disease in patients with clinically negative nodes, for whom such therapies may confer significant survival advantage.^6–9^ Furthermore, SNB-proven micrometastasis may confer eligibility for further clinical trials in this patient group.^2,7^ However, the procedure is not without associated risk, and it is equally important to avoid unnecessary procedures and associated morbidity in patients in whom the likelihood of nodal involvement is truly low.

According to internationally congruent guidelines, SNB is recommended in patients for whom the risk of nodal positivity is > 5%.^6^ However, AJCC stage criteria, for which Breslow’s thickness (BT) and ulceration status form the backbone, are inadequate to accurately stratify patients when used alone.^10^ This is best illustrated in patients with tumors < 2 mm thick (pT1a-pT2a), where some subgroups of T1b have positivity rates < 5%, while some T1a patients have rates > 5%.^11–13^ Beyond the scope of the current staging system, numerous clinicopathologic features have demonstrated associations with SN positivity, including younger age, female sex, presence of mitotic figures, microsatellites, lymphovascular invasion (LVI), absence of tumor-infiltrating lymphocytes, absence of tumor regression, nodular histology, vertical growth phase, and Clark’s level.^14–20^ However, the predictive value of these for SN metastasis in the literature is variable and inconsistent.

Therefore, patient selection for SNB may now need to be reappraised, and as such there has been a renewed drive to identify novel markers that enable the accurate stratification of patients according to their risk of having SN metastasis. BT represents only a one-dimensional surrogate marker for primary tumor burden, however, it is plausible that a histological marker that accounts for the two-dimensional nature of tissue sections could more accurately reflect true tumor burden and therefore prognosis. One such two-dimensional marker, calculated tumor area (CTA), has been previously described and demonstrated to have independent prognostic value and is potentially more powerful than BT as a prognostic feature.^21^

In this work we devised a novel two-dimensional marker, the simplified Breslow area (SBA), and aimed to validate both CTA and SBA as prognostic markers for positive SN status.

## Materials and Methods

A retrospective cohort was collated and analyzed from a single, academic cancer center in the East of England with a prospectively maintained melanoma database. All adult patients diagnosed with primary cutaneous melanoma, with complete primary tumor and outcomes datasets, who were referred for SNB between 2011 and 2014, were included. Patients were excluded if there were multiple or mucosal melanoma primaries, or if the hematoxylin and eosin-stained histopathology slides were unavailable. The research protocol was approved by the Faculty of Medicine and Health Sciences Research and Ethics Committee at the University of East Anglia, including the use of human tissue under the Norwich Research Park Biorepository ethical approval (REC ref.: 19/EE/0089).

Standard patient demographic data and tumor characteristics were recorded. Survival outcomes data including overall survival (OS), disease-specific survival (DSS), and distant metastasis-free survival (DMFS) were also collected. In the case of multiple sites of recurrence, disease-free survival (DFS) was recorded on the basis of the first instance and highest stage at that time, according to the “first/worst” principle. Systemic therapy was offered to eligible patients in accordance with national guidance.

### Statistical Analysis

Statistical analysis of the data was performed using Jamovi software (version 2.3; Sydney, Australia; https://www.jamovi.org). Descriptive statistics was used to summarize patient and primary tumor characteristics stratified by SN status. Medians with interquartile ranges (IQR) were given for continuous variables, while for categorical variables, frequencies with proportions were used. The Kruskal–Wallis and *χ*^2^ tests were used to assess for differences in the medians and proportions, respectively.

**Fig. 1:**
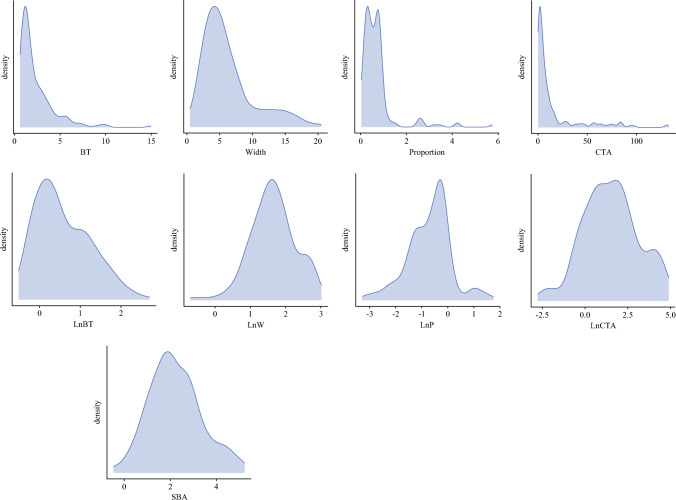
Frequency density plots illustrating the skew of tumor measurements including routinely collected. Breslow thickness (BT), but also invasive width (IW) and calculated tumor area (CTA). The natural logarithmic transformations of CTA (lnCTA) as well as the sum of transformations yielded (lnBT + lnIw = SBA), a novel biomarker which we have termed the Simplified Breslow Area, is shown to be significantly more normally distributed

**Fig. 2:**
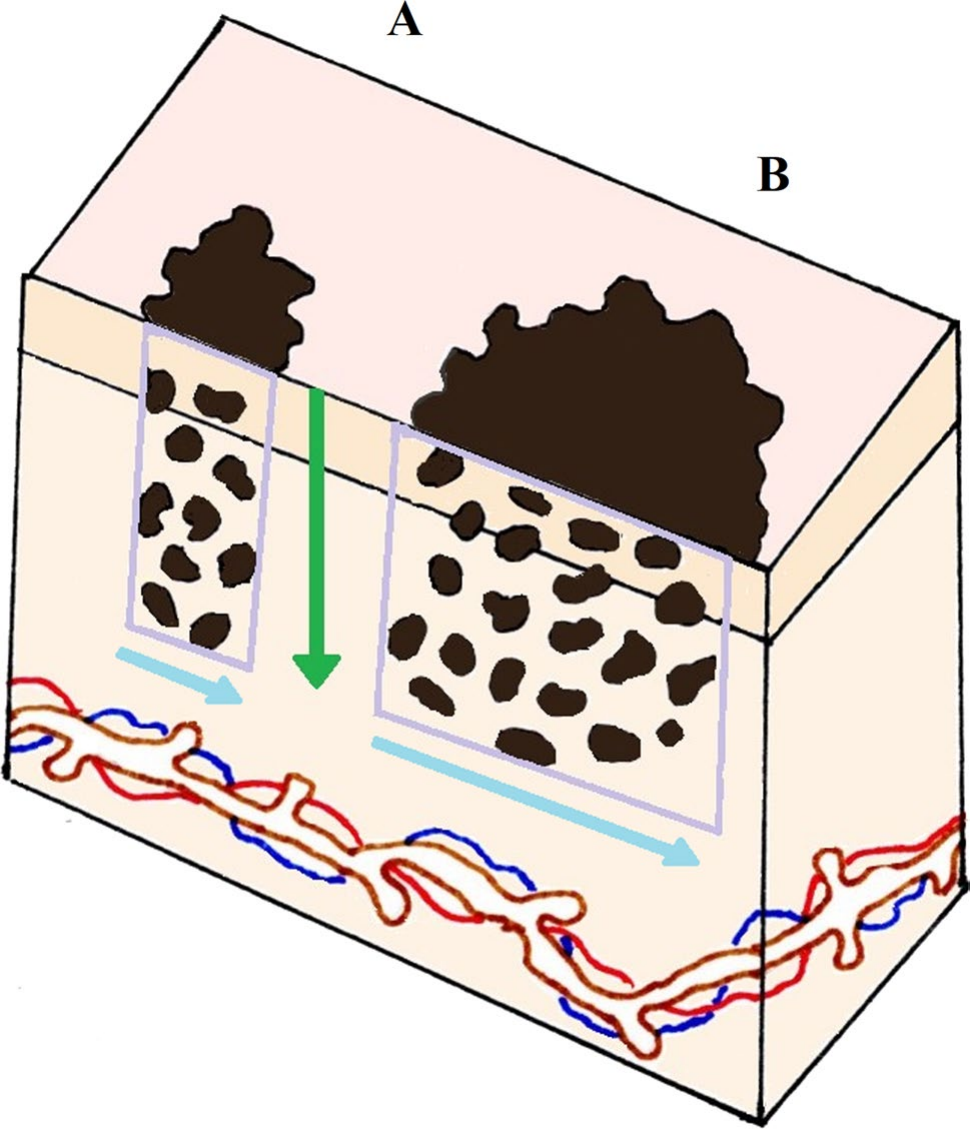
Two stylized examples of melanomas A & B with equal Breslow Thickness (BT) are shown. The calculated tumor area (CTA) box for each tumor, which includes all invasive melanoma cells (brown) within the full tumor breadth on the section with maximal BT, is shown in purple. CTA was calculated by multiplying each tumor’s BT (green arrow), invasive width (IW) (blue arrows) and the proportion of the box occupied by invasive melanoma cells. The simplified Breslow area (SBA) is the natural logarithmic transformation of the product of BT and IW

The distribution of measurements for BT, invasive width (IW) of the primary tumor, and consequently CTA are positively skewed. To achieve a more normally distributed sample with constant variance to facilitate linear regression analysis, these measurements were transformed using the natural logarithm to give lnBT, lnIW, and lnCTA, respectively (Fig. 1). The semiquantitative nature of CTA has been criticized for potentially introducing interobserver variability.^22^ Therefore, in addition to validating CTA against SN status, we sought to test whether an objective quantitative two-dimensional prognostic biomarker incorporating BT and IW may better predict both disease-specific survival and SN status. The sum of their transformations yielded the simplified Breslow area (lnBT + lnIw = SBA) (Fig. 2).

The Kaplan–Meier log-rank method was used to analyze all survival outcomes and differences between groups. The maximally selected rank method was used to study the relationship between lnCTA and SBA, as surrogate markers for tumor burden, and survival outcomes to identify a potential cutoff point that may be used to stratify patients into “high” and “low” risk of SN metastasis. This method investigates all possible cutoff points in the maximum value of lnCTA and SBA, respectively, and identifies the cutoff point value for each marker that achieves the maximum dichotomous separation of the Kaplan–Meier curves into high and low risk groups.^23^ This method was applied to DSS, DFS, and DMFS individually. Once identified for both markers, Cox regressions were then performed to evaluate whether the optimal cutoff point remains significantly associated with outcomes after adjusting for potential confounding factors.

The predictive value of SBA and CTA for SN status was evaluated using separate multivariate logistic regression analyses.

## Results

### Standard Patient and Tumor Characteristics

Of a total cohort of 588 eligible patients, we were able to retrieve slide sets for 177 (30.1%) patients with corresponding SNBs for evaluation. The distribution of standard patient and tumor characteristics collected, stratified according to SN status, is presented in Table 1.

**Table 1:** Cross table of clinicopathologic characteristics of cohort stratified by SN status

Characteristic		SN negative (*n* = 145)	SN positive (*n* = 32)	Test statistic
Age at diagnosis, median (IQR)		65 (53–71)	58 (48–67)	H = 3.25 *p* = 0.07^a^
Male sex		69 (48)	17 (53)	*χ*^2^(1) = 0.32 *p* = 0.57^b^
Primary site	Head and neck	21 (14)	1 (3)	*χ*^2^(3) = 5.56 *p* = 0.13^b^
	Torso	54 (37)	17 (53)	
	Upper limb	36 (25)	5 (16)	
	Lower limb	34 (23)	9 (28)	
Subtype	SSM	107 (74)	27 (84)	*χ*^2^(4) = 3.30 *p* = 0.51^b^
	Nodular	25 (17)	5 (16)	
	Lentigo maligna	8 (6)	0	
	Acral	2 (1)	0	
	Other	3 (2)	0	
AJCC stage^c^	IB	93 (64)	11 (34)	*χ*^2^(3) = 19.69 *p* < 0.01^b^
	IIA	21 (14)	9 (28)	
	IIB	24 (17)	4 (12)	
	IIC	7 (5)	8 (25)	
Lymphovascular invasion		8 (6)	7 (23)	*χ*^2^(1) = 9.23 *p* < 0.01^b^
Ulceration		31 (21)	12 (38)	*χ*^2^(1) = 3.70 *p* = 0.05^b^
Mitotic rate, median (IQR)		3 (1–6)	3 (2–6)	H = 0.37 *p* = 0.54^a^
BT, median (IQR)		1.4 (1.0–2.7)	2.4 (1.3–4.3)	H = 7.39 *p* = 0.01^a^
IW, median (IQR)		4.9 (3.2–7.0)	7.2 (4.6–9.8)	H = 10.57 *p* < 0.01^a^
CTA, median (IQR)		3.2 (1.2–10.9)	6.7 (4.2–26.9)	H = 8.03 *p* = 0.01^a^

Data are expressed as *n* (%) unless otherwise specified. Data on mitotic rate was available for 175 patients and lymphovascular invasion for 173 patients

^a^Kruskal–Wallis with 1 degree of freedom

^b^Pearson (degrees of freedom)

^c^AJCC 8 th edition

*SN* sentinel node, *IQR* interquartile range, *SSM* superficial spreading melanoma, *AJCC* American Joint Committee on Cancer, *BT* Breslow thickness, *IW* invasive width, *CTA* calculated tumor area

The incidence of SN metastasis in our cohort was 18.1% (32/177). Age, primary tumor AJCC stage, lymphovascular invasion, BT, IW, and CTA were all associated with SN status (Table 1). The median BT for the whole cohort was 1.60 mm (IQR 1.10–3.00 mm) and there was significant correlation between BT and the IW of the primary tumor (*R* = 0.527; *p* < 0.001). On multivariate analysis, using logistic regression, we found that of the standard primary tumor characteristics, Breslow thickness, and lymphovascular invasion were the only significant independent predictors of positive SN status.

### Calculated Tumor Area, Simplified Breslow Area, and SN status

The median CTA for SN−patients was 3.2 mm^2^ (IQR1.2–10.9) compared with 6.7 mm^2^ (IQR 4.2–26.9) for SN + patients (*p* < 0.01). The median SBA for SN−patients was 2.0 (IQR 1.3–2.7) compared with 2.8 (IQR 2.1–3.6) for SN+patients (*p* < 0.01).

**Fig. 3:**
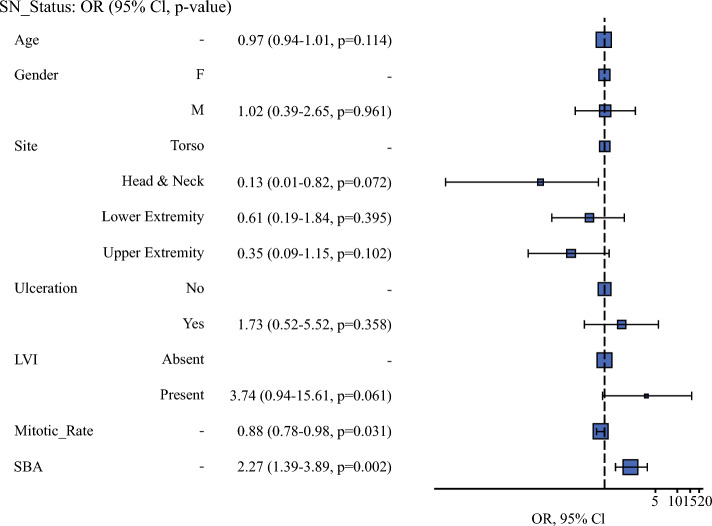
Odds ratio plot from multivariate logistic regression analysis for simplified Breslow area as a predictor of sentinel node status (OR = odds ratio; F = female; LVI = lymphovascular invasion; SBA = simplified Breslow area)

We assessed whether BT, CTA, and SBA were predictors of SN status in separate multivariable logistic regression models each controlling for age, sex, primary tumor site, US, and LVI. Both BT (OR 1.33, 95% CI 1.03–1.71, *p* = 0.023) and SBA (OR 2.27, 95% CI 1.39–3.89, *p* = 0.002) were found to be significant independent predictors of SN status in their respective models. The C-statistic for the SBA model was 79.4%, compared with 77% for the model incorporating BT. In contrast, lnCTA was not found to be an independent predictor of SN status (OR 1.01, 95% CI 0.99–1.03, *p* = 0.293). We found SBA to be the single most important significant independent predictor of SN status (Fig. 3); when SBA (OR 2.65, 95% CI 1.25–6.13, *p* = 0.016) was included in the model with BT, BT was no longer statistically significant (OR 0.90, 95% CI 0.57–1.34, *p* = 0.616) showing significant confounding by SBA.

### Survival

**Fig. 4:**
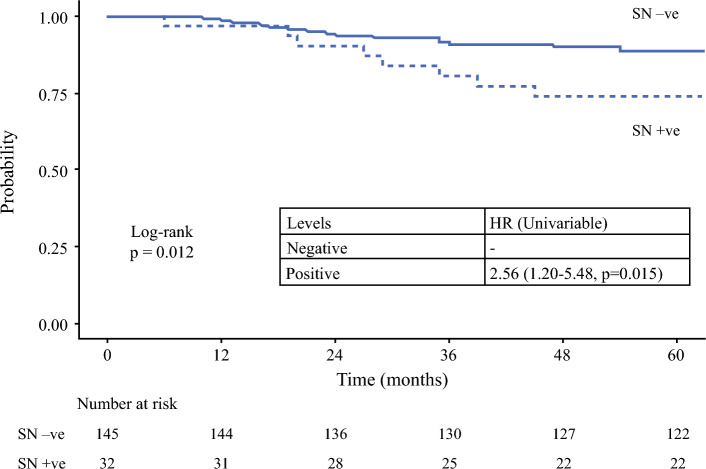
Kaplan–Meier curve for disease specific survival stratified by SN status (SN = sentinel node; HR = hazard ratio)

The median follow-up period was 94 months (IQR 82–106 months). SN positivity was associated with worse DSS, with a 5-year survival of 74.0% compared with 88.8% for SN negative patients (hazard ratio 2.56, 95% confidence interval 1.20–5.48; *p* = 0.015) (Fig. 4).

**Fig. 5:**
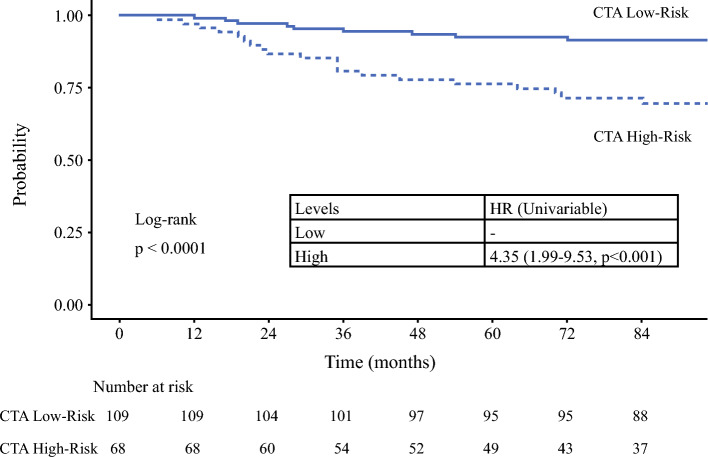
Kaplan–Meier curve for disease specific survival stratified by CTA risk group as determined by MSR statistics (CTA = calculated tumor area; HR = hazard ratio; MSR maximally selected rank)

The maximally selected rank (MSR) method was used to identify optimal cutoff point values for both lnCTA and SBA that would differentiate patients into “high” and “low” risk groups for SN positivity.^23^ MSR analysis identified primaries with a lnCTA of less than 1.84 as low risk, and those greater than 1.84 as high risk, with a 5-year DSS of 86.2% for the low-risk cohort and 68.1% for the high-risk cohort (HR 2.6, 95% CI 1.41–4.79; *p* = 0.002) (Fig. 5). Similar results were seen for DFS and DMFS (Supplementary material). The median OS and DSS of patients in either group was not reached.

**Fig. 6:**
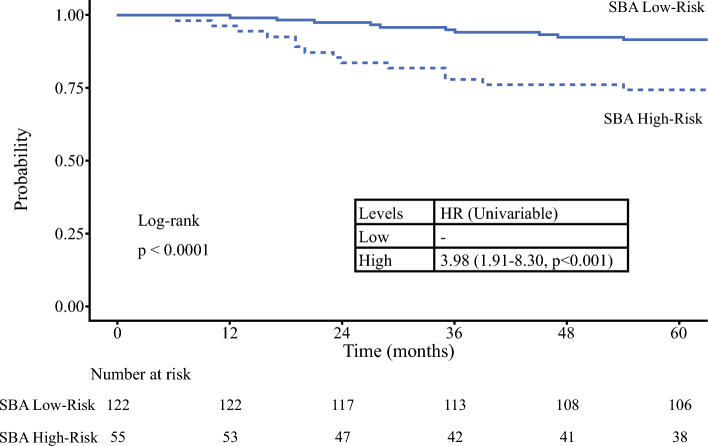
Kaplan–Meier curve for disease specific survival stratified by SBA risk group as determined by MSR statistics (SBA = simplified Breslow area; HR = hazard ratio; MSR maximally selected rank)

The optimum cutoff point according to SBA, determined by the same method, was 2.61 for the whole cohort, with 5-year DSS of 91.6% for the low-risk cohort (i.e., patients whose primaries where SBA was less than 2.61) and 74.4% for the high-risk cohort (i.e., patients with primaries with SBA greater than 2.61) (HR 3.98, 95% CI 1.91–8.30; *p* < 0.001) (Fig. 6). Once again, similar results were seen for DFS and DMFS (Supplementary material). The median OS and DSS of patients in either group was not reached.

The incidence of SN positivity in the low-risk group according to SBA was 12.3% (15/122) compared with 30.9% (17/55) in the high-risk group. Within the low-risk SBA cohort, the median age for SN+patients was 55 (IQR 48–68) compared with 64 (IQR 52–70) for those who were SN−, although again age was not found to be significantly associated with SN status (*p* = 0.28).

When analyzed using Cox proportional hazards regression models as continuous variables, SBA and lnCTA outperformed BT as a prognostic indicator for both DFS (SBA HR 1.62, 95% CI 1.27–2.07, *p* < 0.001; lnCTA 1.47, 95% CI 1.21–1.77, *p* < 0.001; BT HR 1.24, 95% CI 1.12–1.37, *p* < 0.001) and DSS (SBA HR 1.85, 95% CI 1.37–2.49, *p* < 0.001; lnCTA 1.63, 95% CI 1.28–2.06, *p* < 0.001; BT HR 1.29, 95% CI 1.14–1.45, *p* < 0.001). Once again, similar results were seen for OS and DMFS.

## Discussion

The results of our single-center analysis align with established literature regarding the prognostic importance of sentinel node status for disease-specific survival in patients with primary cutaneous melanoma.^24^ However, not all patients benefit from the procedure, and optimal patient selection for SNB continues to be challenging given the limited ability to predict SN positivity, particularly in putative low-risk melanomas such as AJCC pT1b and pT2a primaries. In particular, there remains a significant absence of additional reliable associated clinicopathologic factors, beyond ulceration status, despite the emergence of numerous nomograms and risk stratification tools.^13,20,25^

The incorporation of molecular prognostic information, in the form of gene expression profile (GEP) or immunohistochemistry (IHC) tests, into stratification models has shown promise for refining patient selection for SNB in the future.^26^ Currently, several prognostic tests are commercially available, and some have been shown to discriminate patients who have a < 5% risk of SNB positivity.^27–30^ The potential of GEP tests to influence clinical decision-making has already been evident in the construction of guidelines for other cancer sites, most notably breast.^31^ However, due to significant methodological limitations in these studies, whether there is enough quality evidence supporting their clinical utility in cutaneous melanoma to justify their substantial cost remains highly controversial.^26,32,33^ The use of GEP tests in cutaneous melanoma still requires further prospective investigation with longer follow-ups, especially in the case of thin melanomas, to validate their prognostic utility. and as such, there continues to exist a need for improved clinicopathological prognostic biomarkers.

In their seminal paper, Breslow et al. described the incidence of both recurrent and metastatic disease to be a function of the width, thickness, and stage of invasion of the primary tumor.^34^ Several studies have since demonstrated that increasing tumor volume is associated with a greater chance of metastasis and subsequently poorer survival outcomes.^35–37^ Recently, Saldanha et al. found that a novel two-dimensional marker, CTA, demonstrated improved prognostic accuracy compared with BT, in 1239 patients diagnosed with primary cutaneous melanoma treated in two UK centers.^21^ Our findings corroborate this work, evidenced by the significant separation of the survival curves in our cohort according to the optimal cutoff point for CTA. Compared with our dataset, cohorts were well matched for sex (male, 47.6% versus 48.6%) and age (60 years, IQR 47–71 years versus 64 years IQR 52–71 years). The tumors analyzed by Saldanha et al. were typically thinner [median BT 0.9 mm (IQR, 0.5–2.0 mm) versus BT 1.60 mm (IQR 1.10–3.00 mm)] with a lower proportion of ulcerated tumors (16% versus 24.2%) and consequently of an earlier AJCC stage at diagnosis (AJCC pathological stage I, 49.7% Norwich versus 73.6% Leicester). Therefore, unsurprisingly, the median CTA for our cohort was also higher [4.40 (IQR 1.6–11.3) versus 1.30 IQR (0.2–6.4)]. The larger proportion of patients with high-risk features in our dataset is representative of the cohort of patients that are selected for SNB in a modern melanoma practice, however, as a single-center analysis, our findings should nonetheless be interpreted with caution bearing in mind the potential for measurement and selection biases.

Our study did not find that CTA was a significant predictor of SN status in univariate or multivariate logistic regression analysis. To our knowledge, only one other group has investigated the predictive value of CTA for SN status. An analysis of 271 patients who underwent SNB between 2004 and 2018 from three centers in the USA found that BT, IW, and CTA had similar areas under a receiver operating characteristic (ROC) curve in separate multivariate logistic regression models adjusted for age, sex, US, mitotic rate, and LVI.^22^ This study investigated a similar size cohort but did not assess survival as an outcome. We hypothesized that these results were likely due to a degree of error introduced by the subjective assessment of proportion of the visual field involved by tumor as part of the CTA measurement, and that this was further exaggerated by the relatively greater proportion of larger tumors in datasets of patients selected for SNB. In the initial validation of CTA, the intraclass correlation coefficient for 13 primary melanoma samples independently scored by two observers was 0.99, however, further study is required to demonstrate that these findings can be replicated by others.^21^

In this work, we describe, for the first time, a more objective factor, namely SBA, which removes this subjective aspect of the assessment (SBA = lnBT + lnIW) and thus may have improved clinical applicability. Subsequent multivariate analysis demonstrated that our novel derived biomarker, SBA, was the single most important predictor of sentinel node status, outperforming LVI, ulceration status, and BT. Importantly, SBA shares the practicality, low cost, and ease of dissemination as CTA, and has the additional advantage that its measurement and calculation can be easily achieved using digital pathology techniques where such software is available. There is evidence that computer-assisted analysis of multidimensional tumor burden may enhance the objectivity and prognostic accuracy further, and we suggest that this may lend itself to automation with artificial intelligence algorithms.^38^

Our findings should be interpreted cautiously in light of several limitations, the most substantial being the potential measurement and selection biases inherent to a single-center study as well as to a relatively small sample size, attested by the wide 95% confidence intervals seen in our analyses. The latter is particularly pertinent in the subsample of patients with pT1b-pT2a tumors (*n* = 96), for whom biomarkers to guide patient selection for SNB will be especially valuable for the construction of international guidelines. Therefore, independent validation of our findings in a prospective study using a large, multi-institutional, international cohort of SNB patients is necessary prior to translation into clinical practice.

## Conclusions

Our study is the first to validate the two-dimensional histological marker, calculated tumor area, against sentinel node status using survival analysis. The transformation of tumor area into a reproducible objective measure, simplified Breslow area, achieved superior prognosis of both sentinel lymph node status and survival in primary invasive melanoma compared with Breslow thickness. This may be enhanced further using digital pathology software, and lends itself to the potential for automation to achieve increased accuracy and objectivity, ultimately improving patient selection for staging with SNB.

## Supplementary Information

Below is the link to the electronic supplementary material.

Supplementary file1 (DOCX 136 kb)
