# Supplementary figures and images for: Validation of Invasive Area for Predicting Sentinel Node Status and Survival in Primary Cutaneous Melanoma
Source: Ann Surg Oncol. 2025 May 23;32(9):6927–35. doi: 10.1245/s10434-025-17442-2 (PMC12317915; doi:10.1245/s10434-025-17442-2)

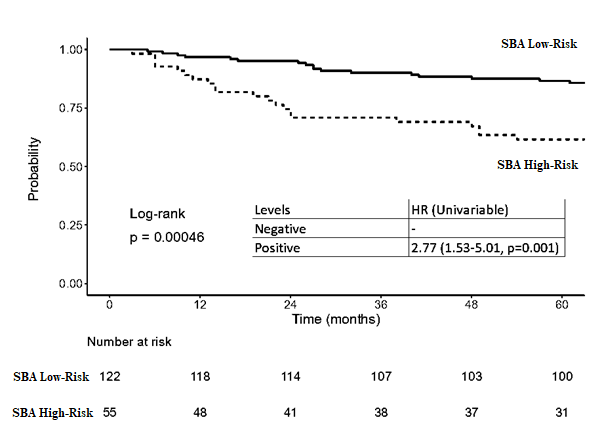

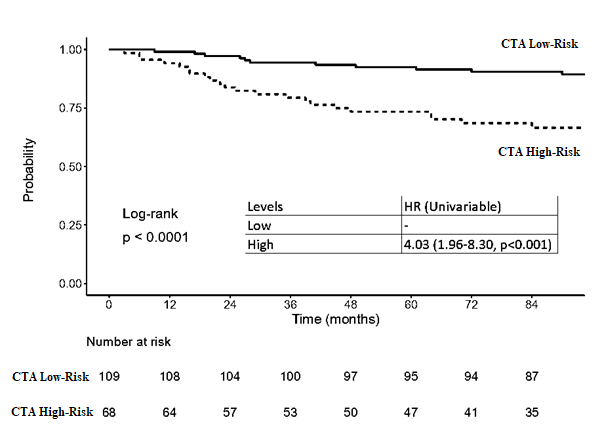

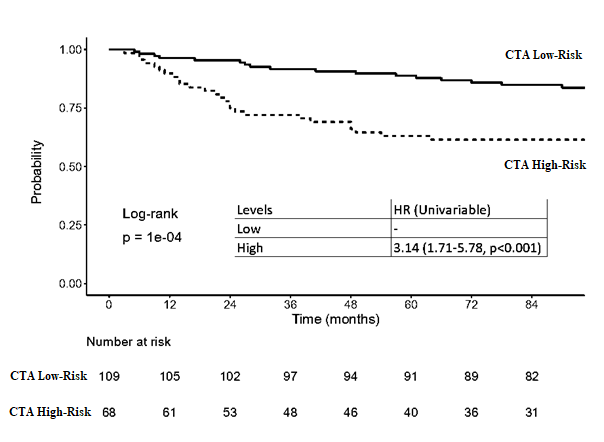

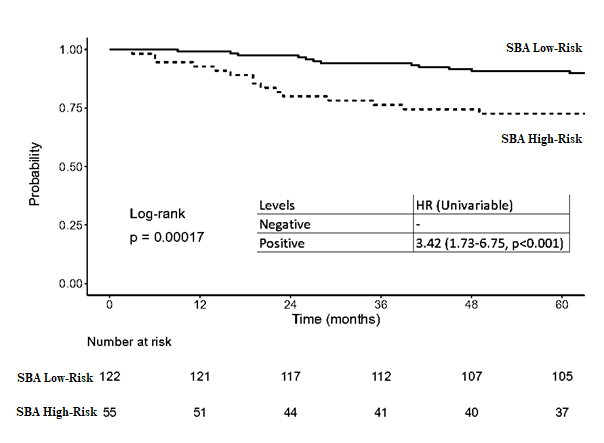

Supplement: Supplementary file 1 — Supplementary file1 (DOCX 136 kb) [file 10434_2025_17442_MOESM1_ESM.docx]
